# A Tertiary Lymphoid Structure–Derived Prognostic Signature Integrates Immune Microenvironment and Mutational Landscapes in Clear Cell Renal Cell Carcinoma

**DOI:** 10.1155/humu/8446751

**Published:** 2026-04-28

**Authors:** Xuanyu Zhou, Zhongwei Zhao, Kai Huang, Guangxin Ma

**Affiliations:** ^1^ Department of Medical Oncology, Qilu Hospital of Shandong University, Jinan, Shandong, China, qiluhospital.com; ^2^ Department of Urology, Qilu Hospital of Shandong University, Jinan, Shandong, China, qiluhospital.com; ^3^ Department of Geriatics, Qilu Hospital of Shandong University, Jinan, Shandong, China, qiluhospital.com

## Abstract

Tertiary lymphoid structures (TLSs) are increasingly recognized as important components of the tumor immune microenvironment, yet their prognostic and immunological implications in clear cell renal cell carcinoma (ccRCC) remain incompletely characterized. In this study, we performed an integrated bioinformatic and translational analysis to investigate TLS‐associated molecular features in ccRCC. Using TCGA‐KIRC transcriptomic data, we identified three TLS‐related molecular subtypes with distinct survival outcomes and immune microenvironment characteristics. Based on prognostic TLS‐associated genes, we developed a four‐gene TLS‐derived score (CSF2, CXCL13, IL1R2, and SGPP2) that stratified patients into groups with significantly different overall survival. The TLS score remained an independent prognostic factor after adjustment for clinical variables. Interestingly, higher TLS scores were associated with increased immune infiltration but poorer survival outcomes, suggesting that TLS‐associated transcriptional patterns may reflect heterogeneous immune functional states rather than uniformly effective antitumor immunity. Computational analyses indicated potential differences in predicted immunotherapy response and mutation landscapes between TLS score groups. Limited experimental validation using fresh ccRCC specimens supported the feasibility of TLS score assessment and provided preliminary histopathological context for TLS‐associated immune features. Overall, this study proposes a TLS‐derived transcriptional signature that may help capture immune heterogeneity in ccRCC and may provide a complementary framework for prognostic assessment. Further studies are required to validate its biological and clinical relevance.

## 1. Introduction

Clear cell renal cell carcinoma (ccRCC) accounts for 70%~80% of all types of renal carcinoma, and its prognosis varies depending on the disease stage [1]. Patients with early‐stage ccRCC who undergo surgery generally have a favorable prognosis, whereas those with advanced, metastatic, or recurrent ccRCC often face poor outcomes due to a lack of effective treatment options [2]. Therefore, a key focus of current research is to further refine ccRCC risk assessment and identify new therapeutic targets [3].

Tertiary lymphoid structures (TLSs) are organized lymphoid aggregates with specialized networks of fibroblasts, sharing many functional and structural characteristics with secondary lymphoid organs (SLOs), including lymph nodes [4]. SLOs throughout the body enable antigen sampling from different tissues, thereby facilitating the induction of adaptive immune responses. Under conditions of persistent chronic inflammation, ectopic lymphoid neogenesis can occur, leading to the formation of TLSs at organ sites [5]. Fibroblasts and several molecules essential for the maintenance and function of SLOs, such as homeostatic chemokines and lymphotoxins, are involved in the formation of TLSs [6]. TLSs play roles in the development and treatment response of various chronic diseases, including autoimmune disorders, chronic infections, and cancers [4].

Due to their capacity to develop outside the lymphatic system, TLSs participate in the tumorigenesis and progression of various cancers [6]. Tumor‐infiltrating lymphocytes (TILs) can be recruited from SLOs into the tumor microenvironment (TME) and supported by TLSs. Antitumor immune responses are initiated within TLSs located adjacent to tumors [7]. Accordingly, in cancers such as colorectal cancer, breast cancer, and hepatocellular carcinoma, the presence of mature TLSs with germinal centers is a positive prognostic indicator, whereas immature TLSs show no or weak correlation with survival [8–10]. Furthermore, the presence of TLSs is associated with improved treatment responses in certain cancer types [11]. Given the role of TLSs in modulating tumor‐specific immune responses, their prognostic and predictive potential has received growing attention. Therapies aimed at inducing or enhancing TLS functionality hold clinical promise for improving tumor control [5].

The role of TLSs in ccRCC remains incompletely understood. In mildly injured kidneys, TLS development halts before germinal center formation, whereas in severely damaged kidneys, TLSs become fully mature, indicating a potential association between TLSs and renal inflammation [12]. In ccRCC, most TLSs are immature, and these densely distributed immature TLSs are associated with poor prognosis [13]. Moreover, the presence of TLSs in pretreatment biopsies of ccRCC has been shown to correlate with response to PD‐1 blockade or combined PD‐1 and cytotoxic T‐lymphocyte‐associated protein 4 (CTLA‐4) blockade [14]. Further exploration of the role of TLSs in ccRCC and their potential as therapeutic targets holds important clinical implications.

In this study, we analyzed the expression profiles and genetic alteration landscape of TLS‐related genes in ccRCC and identified ccRCC molecular subtypes based on these genes. After characterizing the immune microenvironment features associated with TLSs in ccRCC, we constructed a prognostic model using a TLS score. Subsequent analyses of gene mutations, immunotherapy response, and drug resistance provide insights for personalized clinical treatment. Our findings offer new perspectives and evidence on the role of TLSs in ccRCC.

## 2. Materials and Methods

### 2.1. Transcriptome Data, Mutation File, and Copy Number Variation (CNV) Collection

The genetic microarray data and clinical information file of each ccRCC sample was downloaded from the TCGA database by searching the project “TCGA‐KIRC.” A total of 611 samples (72 normal samples and 539 ccRCC samples) were collected from the TCGA database, and Perl language was used to extract the expression matrix of each sample. Based on human gene annotated reference, the gene symbol was annotated utilizing Perl language. The genetic mutation file and CNV file of ccRCC were downloaded from the UCSC Xena database, and Perl script was used for preprocessing the files.

### 2.2. Collection of TLS‐Related Genes and Differential Expression Analysis

The list of TLS‐related genes was identified from the Cabrita et al.′s research, and 39 TLS‐related genes were collected [15]. The “limma” package was utilized to examine the expression difference of TLS in normal and tumor tissues, and the “RCircos” package was used to locate the position of TLS in chromosome. The interaction (PPI) of TLS was explored using STRING database.

### 2.3. Comprehensive Analysis of TLS‐Related Molecular Subtypes

Integrating the survival status and TLS expression matrix of ccRCC, the prognostic value (hazard ratio [HR] and*p* value) of TLS was estimated utilizing the LASSO‐univariate Cox analysis. Then, multivariate Cox analysis was employed to select the independent prognosis signature for ccRCC. On the basis of ccRCC prognostic signature, the script “ConsensusClusterPlus” was carried out to identify the TLS‐related molecular subtypes for ccRCC. Principal component analysis (PCA) was employed to exhibit the distribution pattern of ccRCC TLS molecular subtypes via “ggplot2” script. Based on the KEGG reference symbol “c2.cp.kegg.v7.2.symbols.gmt,” the GSVA algorithm was developed to examine the difference of KEGG signaling pathways in TLS molecular subtypes.

### 2.4. Immune Microenvironment Characterization Evaluation and Immunotherapy Response Prediction

Referring the marker genes of each immune cell, a single sample gene set enrichment analysis (ssGSEA) was developed to evaluate the 23 immune cells infiltration status of ccRCC based on the gene expression matrix. The immune status was evaluated using “estimate” script. According to the transcriptomic biomarkers, the Tumor Immune Dysfunction and Exclusion (TIDE) was estimated to predict the immunotherapy response of ccRCC samples. Moreover, the comprehensive immunogenomic analyses of ccRCC were conducted using The Cancer Immunome Atlas (TCIA) database.

### 2.5. Estimation of TLS Score and Prognostic Model Establishment

According the TLS prognostic signature, the TLS score of each ccRCC sample was estimated: TLS score = TLS prognostic signature (i) ∗ coefficient (i). “caret” script was utilized to classify the ccRCC samples into training and validation sets under the classification threshold of 7:3. Based on the cutoff of survival time, the ccRCC samples in the entire training and test sets were classified into low and high TLS score prognostic model.

### 2.6. Clinical Characteristic Feature and Independent Prognosis Analysis

The “ComplexHeatmap” script was used to exhibit the TLS score in each ccRCC sample of different clinical characteristic features. Integrating the clinical feature information and TLS score, we used the univariate and multivariate Cox analysis to estimate the HR and *p* value of the variables. Based on the clinical feature and TLS score, we used the “rms” to develop a nomogram plot to estimate the 1‐, 3‐, and 5‐year survival probability of ccRCC samples and used “regplot” to evaluate the consistency of nomogram predicted clinical outcome and the actual clinical outcome.

### 2.7. Genetic Mutation Landscape and Drug Sensitivity Prediction

The genetic mutation files of ccRCC were downloaded from the TCGA database, and the Perl language was utilized to prepare the waterfall file. “maftools” script was employed to visualize the genetic mutation frequency of TLS score subgroups. The drug sensitivity prediction was carried out based on the Genomics of Drug Sensitivity in Cancer (GDSC) database via “pRRophetic” script.

### 2.8. Statistical Analysis

The log‐rank algorithm was utilized to estimate the clinical survival outcome of ccRCC samples. The data processing and analysis were all conducted in R soft and Perl language environment. The Wilcoxon rank‐sum test was used to examine the significance level between two groups, and ANOVA test was employed to examine the significance level between three groups. *p* < 0.05 was considered statistically different. Statistical analysis of the histological experiment section was performed using the GraphPad Prism software (Version 9.0). Differences in cell mortality rates between the high and low TLS score groups were compared using an unpaired, two‐tailed Student′s *t*‐test. A *p* value of < 0.05 was considered statistically significant. The correlation between protein expression levels of characteristic genes within TLS‐adjacent areas and CD8+ or CD4+ T‐cell infiltration was assessed by Pearson/Spearman correlation analysis.

### 2.9. Clinical Sample Collection

A total of 10 fresh ccRCC tumor tissue samples were collected from patients undergoing radical nephrectomy. The acquisition of all samples was conducted with patient informed consent and approved by the Institutional Ethics Committee of our hospital (Approval No.: KYLL‐2024(ZM)‐058). Immediately after collection, nine samples were aseptically divided into three portions: one portion was snap‐frozen in liquid nitrogen for subsequent RNA extraction, one portion was fixed in 4% paraformaldehyde for paraffin embedding and immunohistochemical (IHC) analysis, and the third portion was used within 2 h for the culture of primary patient‐derived tumor cells (PDTFs). One sample was excluded from this division due to insufficient tissue volume and was used only for RNA extraction and subsequent quantitative reverse transcription polymerase chain reaction (qRT‐PCR) analysis.

### 2.10. RNA Extraction, qRT‐PCR, and TLS Score Calculation

Total RNA was extracted from the frozen tissues of all 10 samples using TRIzol reagent (Invitrogen, United States). RNA concentration and purity were measured using a NanoDrop spectrophotometer. Reverse transcription to synthesize cDNA was performed using the PrimeScript RT reagent Kit (Takara, Japan). The mRNA expression levels of four TLS signature genes (CSF2, CXCL13, IL1R2, and SGPP2) were quantified using SYBR Green Premix (Takara, Japan) on a QuantStudio 5 Real‐Time PCR System (Applied Biosystems, United States), with GAPDH serving as the internal reference gene. The relative expression of each gene was calculated using the 2^(−*ΔΔ*Ct) method. The TLS score for each sample was calculated according to the established formula: TLS Score = (relative expression of CSF2 × 0.592) + (relative expression of CXCL13 × 0.104) + (relative expression of IL1R2 × 0.104) + (relative expression of SGPP2 × (−0.325)). All samples were ranked based on their TLS scores. The optimal cutoff value for stratifying patients into high and low TLS score groups was determined using maximally selected rank statistics implemented in the R package “survminer.”

### 2.11. PDTF Culture and In Vitro Drug Sensitivity Assay

Primary PDTF cultures were established from nine fresh tumor samples using the tissue fragment method. Briefly, tissues were minced into appropriately sized fragments, mixed with Matrigel, and plated at the bottom of 96‐well plates. Cultures were maintained in DMEM high‐glucose medium supplemented with 10% fetal bovine serum (FBS). During the culture process, three cases were excluded due to culture failure or poor cell growth. Consequently, six successfully established PDTF lines with good growth status were included in the subsequent drug sensitivity assay.

To simulate first‐line clinical combination immunotherapy, the cultures were treated for 72 h with a combination of anti‐PD‐1 (Nivolumab, 10 *μ*g/mL) and anti‐CTLA‐4 (Ipilimumab, 10 *μ*g/mL) antibodies. The control group was treated with an equivalent concentration of isotype control IgG.

After treatment, cell viability was assessed using a live/dead cell assay kit (Calcein‐AM for live cells and propidium iodide [PI] for dead cells; Proteintech, PF00008) according to the manufacturer′s instructions. Fluorescence intensity was quantified using a fluorescence microplate reader. Cell mortality was calculated as the ratio of dead cell fluorescence intensity to total fluorescence intensity.

### 2.12. Histological Staining (Hematoxylin and Eosin [H&E], Immunohistochemistry [IHC], and Immunofluorescence [IF])

Formalin‐fixed, paraffin‐embedded (FFPE) tissue blocks from the six selected samples were sectioned consecutively at a thickness of 4 *μ*m.

H&E staining: H&E staining was performed first. The stained sections were examined by an experienced pathologist under a light microscope to identify regions containing TLS.

IF: Consecutive sections adjacent to the H&E‐identified regions were used for IF staining. Following antigen retrieval, sections were incubated overnight at 4°C with primary antibodies against CD4 and CD8 (CD4 antibody, Proteintech, 67786‐1‐Ig; CD8 antibody, Proteintech, 66868‐1‐Ig; both diluted at 1:1000). This was followed by incubation with corresponding Alexa Fluor 488– and 594–conjugated secondary antibodies. Nuclei were counterstained with DAPI. Images were acquired using a confocal microscope to visualize T lymphocyte infiltration within the TLS regions.

IHC: Consecutive sections adjacent to those used for IF were subjected to IHC staining. After antigen retrieval, sections were incubated overnight at 4°C with primary antibodies against CSF2, CD200, and IL1R2 (CSF2 antibody, Proteintech, 17762‐1‐AP; CD200 antibody, Proteintech, 66282‐1‐Ig; IL1R2 antibody, Proteintech, 60262‐1‐Ig; all diluted at 1:1000). Subsequently, HRP‐conjugated secondary antibodies were applied, and staining was visualized using DAB substrate. Nuclei were counterstained with hematoxylin. Protein expression differences of these signature genes in the peri‐TLS areas were compared between the high and low TLS score groups under a microscope.

### 2.13. Primer Design and Synthesis

The primer sequences used in this study were designed based on the coding sequences, utilizing the NCBI Primer Designing Tool (https://www.ncbi.nlm.nih.gov/tools/primer-blast/). Primer specificity was verified using the PrimerBank database (https://pga.mgh.harvard.edu/primerbank/). All primers were synthesized by Sangon Biotech (Shanghai) Co. Ltd. and purified by PAGE. Primer dry powders were centrifuged, dissolved in sterile nuclease‐free water to prepare 100‐*μ*M stock solutions, and stored at −20°C. Working solutions at 10 *μ*M were prepared by diluting the stock solutions with nuclease‐free water immediately before use. The specific primer sequences used are listed in Table 1.

**TABLE 1:** Primer sequences used for quantitative real‐time PCR in this study.

Gene name	Forward primer sequence 5^′^⟶3^′^	Reverse primer sequence 5^′^⟶3^′^
GAPDH	GAGAAGGCTGGGGCTCATTT	AGTGATGGCATGGACTGTGG
CSF2	TAATCTAGGGGGTGGGGTCG	GAGTGCCTGAGGTCCTTGTC
SGPP2	TGTCTCAGCAGACTCTACAC	GAGTCCAGGGAATCGATGAG
CXCL13	CTCTCCAGGCCACGGTATT	TAACCATTTGGCACGAGGAT
IL1R2	AGTGTGCCCTGACCTGAAAGA	TCCAAGAGTATGGCGCCCT

## 3. Results

### 3.1. Expression Characteristic and Genetic Variation Landscape of TLS in ccRCC

We collected 39 TLS‐related genes to explore their potential function in the tumorigenesis of ccRCC. Utilizing the “limma” script, the expression characteristic of TLS in ccRCC was determined, and the result illustrated a significant difference of most TLS‐related genes in normal and tumor tissues (Figure 1A). The frequency of CNV demonstrated that CSF2 showed a remarkable CNV amplification, whereas SGPP2, CCL20, PDCD1, and CCR5 displayed a clear CNV deletion in ccRCC and PPI network revealed a close interaction between 39 TLS‐related genes (Figure 1B).

FIGURE 1Landscape of TLS‐related genes and derivation of molecular subtypes in ccRCC. (A) The differential expression analysis of 39 TLS‐related genes in normal and tumor tissues. (B) CNV frequency analysis of TLS and protein–protein interaction analysis between 39 TLS‐related genes. Significance level: ∗*p* < 0.05, ∗∗*p* < 0.01, ∗∗∗*p* < 0.001. ns: no significance. (C, D) LASSO–univariate Cox analysis for selecting characteristic prognosis variables. (E) PCA diagram shows the classification independence between TLS cluster A, B, and C. (F) Clinical outcome analysis of ccRCC in TLS cluster A, B, and C. (G) TLS expression characterization in TLS molecular subtypes and clinical variables.

**Figure:**
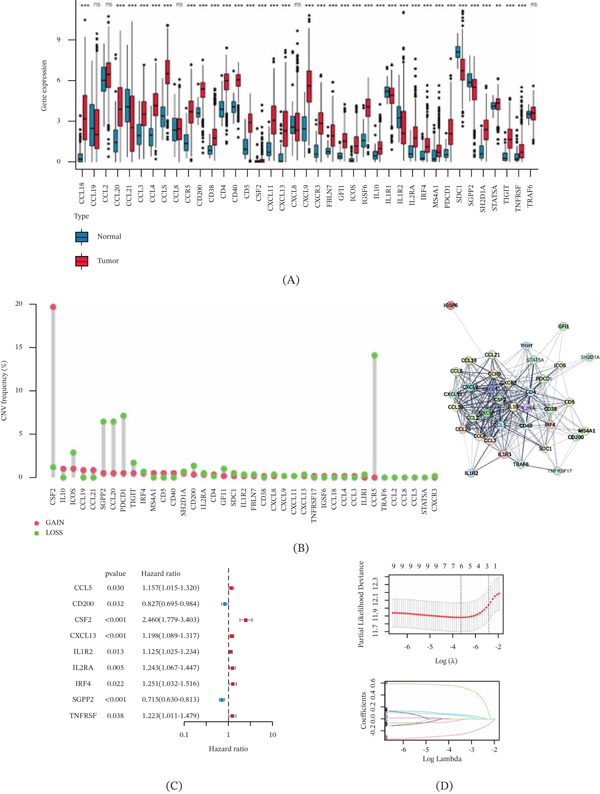

**Figure:**
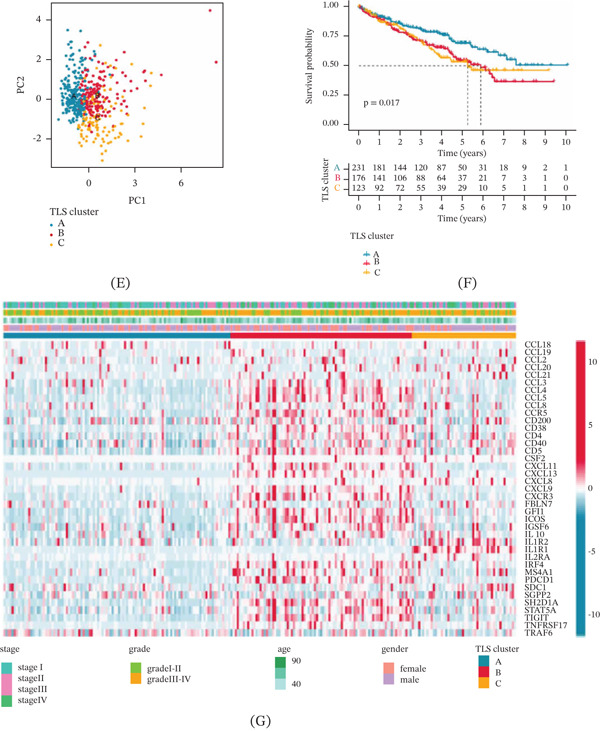

### 3.2. TLS‐Based Molecular Subtypes Identification of ccRCC

Based on the LASSO–univariate Cox analysis, the prognosis value of TLS for ccRCC was evaluated, and the results identified nine prognostic TLS in total, with two favorable factors (CD200 and SGPP2) and seven risk favorable factors (CCL5, CSF2, CXCL13, IL1R2, IL2RA, IRF4, and TNFRSF17) (Figure 1C). Five characteristic variables were selected via LASSO analysis, and four independent prognostic variables were obtained using multivariate Cox analysis (Figure 1D). Utilizing the expression of TLS prognostic signature, we performed an unsupervised consensus clustering to cluster the ccRCC samples into different molecular subtypes (cluster A: 231, cluster B: 176, cluster C: 123). The PCA diagram demonstrated an independent distinction between TLS cluster A, B, and C (Figure 1E). Survival outcome analysis suggested that the clinical prognosis of ccRCC samples in TLS cluster A was better than TLC cluster B and C (Figure 1F *p* = 0.017). The heat map illustrated the correlation between TLS expression, TLS subtypes, and clinical variables, and the ccRCC samples with better prognostic outcome in TLS cluster A were associated with lower expression of TLS‐related genes (Figure 1G).

### 3.3. Immune Microenvironment Characterization of ccRCC in TLS Cluster Subtypes

**FIGURE 2:**
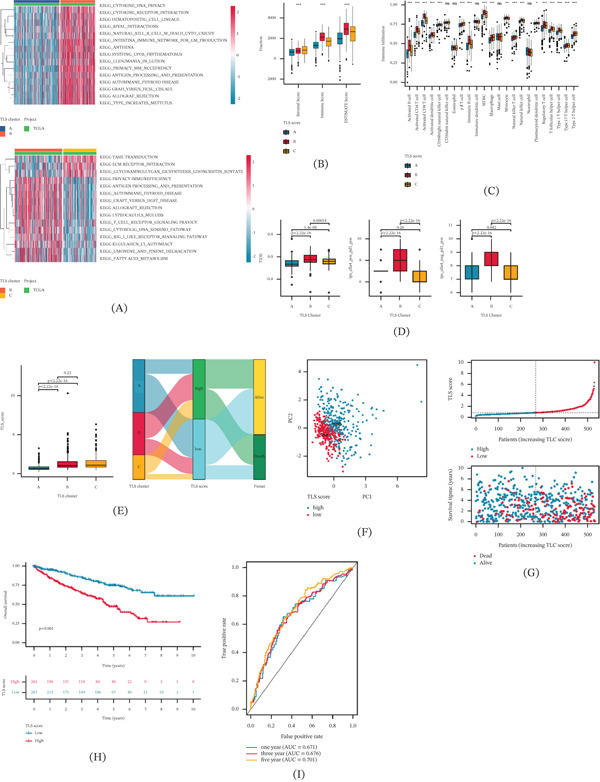
Functional characterization of subtypes and construction of the TLS score. (A) GSVA shows the KEGG terms between TLS cluster subtypes. (B) Estimation algorithm of immune, stromal, and estimate scores. (C) The immune infiltration proportion of immune cells in TLS cluster subtypes. (D) Immunotherapy response prediction of TLS cluster subtypes. Significance level: ∗*p* < 0.05, ∗∗*p* < 0.01, ∗∗∗*p* < 0.001. ns: no significance. (E) Difference analysis of TLS score in TLS cluster subtypes and Sankey diagram shows the relationship of TLS cluster, TLS score, and clinical outcome. (F) PCA analysis of TLS score subtypes. (G) Division of TLS score subtypes. (H) Clinical OS outcome analysis of ccRCC in TLS score subtypes. (I) The time‐dependent ROC analysis (1, 3, and 5 years).

To better understand the potential molecular mechanism of ccRCC samples in TLS cluster subtypes, we utilized GSVA algorithm to estimate the potential regulation KEGG pathways in ccRCC. Compared to TLS cluster A, we observed that immune‐related signaling pathways were upregulated in TLS cluster B, including cytokine–cytokine receptor interaction, natural killer cell‐mediated cytotoxicity, and intestinal immune network for IgA production. Between TLS cluster B and C, we also found that some immune‐related signaling pathways were greatly downregulated in TLS cluster C (Figure 2A). Based on the GVA analysis, we further evaluated the immune microenvironment characterization of TLS cluster subtypes, and a notable difference was observed in the immune status of TLS cluster subtypes. The ccRCC samples with worse prognosis outcome in TLS cluster B were associated with higher immune and estimate scores (Figure 2B). Utilizing the ssGSEA estimation algorithm, we further investigated the immune infiltration landscape of TLS cluster subtypes, and the estimation result suggested that the TLS cluster B had higher immune infiltration of most immune cells, such as activated B cell, CD4^+^ T cell, and CD8^+^ T cell, indicating a higher immune status of ccRCC in TLS cluster B (Figure 2C). Notably, immune response prediction results demonstrated that the TIDE score of TLS cluster A was lower than TLS cluster B and C, implying a better immunotherapy response of ccRCC in TLS cluster A. IPS analysis revealed that the TLS cluster B may respond better to the treatment of CTLA4 and PD‐1 (Figure 2D). In summary, we conclude that the TLS‐based molecular subtypes could distinguish the clinical survival outcome and immune infiltration characterization and also could indicate the immunotherapy response to CTLA4 and PD1 for ccRCC samples.

### 3.4. Development of TLS Score to Establish a Prognostic Model for ccRCC

Utilizing the TLS prognostic signatures, the TLS score of each ccRCC samples was estimated according to the calculation formula: TLS score = CSF2∗0.592 +  CXCL13∗0.104  +  IL1R2∗0.104  +  SGPP2∗− 0.325. In the TLS cluster, we found that the ccRCC samples in TLS cluster A with better clinical OS outcome were associated with lower TLS score. The Sankey diagram exhibited that relationship of TLS score, TLS cluster, and clinical survival outcome (Figure 2E). According to the optimal division of TLS score, the ccRCC samples were classified into low and high TLS score subtypes, and the PCA plot revealed a remarkable distinction between TLS score subtypes (Figure 2F). Meanwhile, we further investigated the association between TLS score and clinical OS outcome for ccRCC, and the scatter plot suggested that most of the death samples were associated with high TLS score and lower survival time (Figure 2G). Clinical survival curve illustrated that the OS outcome of ccRCC samples with low TLS score was substantively better than high TLS score subtypes (Figure 2H, *p* < 0.001). Time‐dependent ROC result showed that the AUC of 1, 3, and 5 years was 0.671, 0.676, and 0.701, respectively (Figure 2I). Based on these results, we hypothesized that the TLS score–based prognostic model constructed of TLS prognostic signature could accurately differentiate the clinical prognosis of ccRCC.

### 3.5. Verification of TLS Score–Based Prognostic Model in Independent Cohorts

**FIGURE 3:**
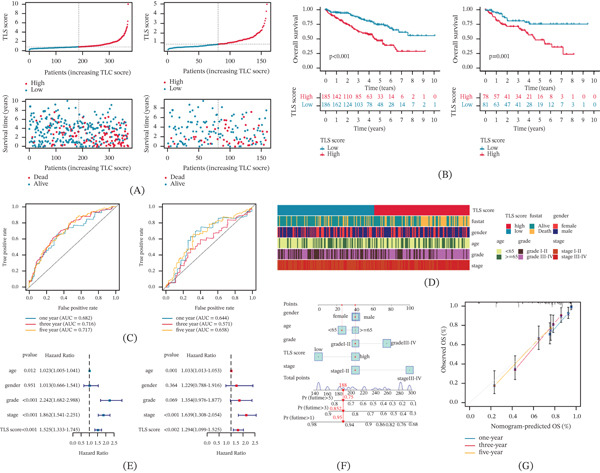
Validation of the TLS score as an independent prognostic biomarker. (A) The classification of TLS score subtypes in training and test sets. (B) Clinical OS outcome analysis of ccRCC with low and high TLS score in both cohorts. (C) ROC analysis of 1, 3, and 5 years in both cohorts. (D) TLS score distribution in different clinical characterization. (E) Univariate and multivariate Cox analysis of TLS score and clinical characterization. (F, G) Nomogram construction and calibration curve evaluation.

Considering the potential prognosis value of TLS score for ccRCC, we classified the ccRCC samples into two independent cohorts to further validate the independence of TLS score in predicting clinical OS outcome. Utilizing the “caret” package, a classification ratio of 7:3 was set to divide the ccRCC samples into training set and test set. According to the TLS prognostic signature, the TLS score of each ccRCC sample was estimated, and the TLS score subtypes were identified (Figure 3A). The clinical OS outcome results in training and test sets illustrated that the clinical prognosis of ccRCC with low TLS score was substantially better than those with high TLS score (Figure 3B). Time‐dependent ROC curve results in the two independent cohorts suggested that the AUC of 1, 3, and 5 years was 0.682, 0.716, 0.717 in training set and 0.644, 0.571, and 0.658 in test set, respectively (Figure 3C). According to the results of these prognostic models, we conclude that the TLS score‐based model can be accurately employed to evaluate the clinical prognosis outcome of ccRCC, and ccRCC with higher TLS score is associated with worse clinical OS outcome.

### 3.6. Comprehensive Analysis of Independent Prognosis of TLS Score and Nomogram Establishment

We further investigated the correlation of TLS score in different clinical characterization of ccRCC. The distribution result of TLS score in clinical characterization suggested that the ccRCC in Grade III–IV and Stage III–IV was associated with higher TLS score (Figure 3D). Univariate Cox analysis of TLS score and different clinical characterization illustrated that age (*p* = 0.012, HR = 1.023(1.005–1.041)), grade (*p* < 0.001, HR = 2.242(1.682–2.988)), stage (*p* < 0.001, HR = 1.862(1.541–2.251)), and TLS score (*p* < 0.001, HR = 1.525(1.333–1.745)) were associated with poor clinical outcome for ccRCC. Multivariate Cox analysis demonstrated that the TLS score (*p* = 0.002, HR = 1.294(1.099–1.525)) was an independent prognosis indicator for ccRCC (Figure 3E). Based on the TLS score and clinical characterization, a nomogram was established to evaluate the 1‐, 3‐, and 5‐year survival probability of ccRCC, and the calibration curve demonstrated an accurate consistency between nomogram predicted OS and actual OS for ccRCC (Figure 3F,G).

### 3.7. Evaluation of Immune Microenvironment and Immunotherapy Response in TLS Score Subtypes

**FIGURE 4:**
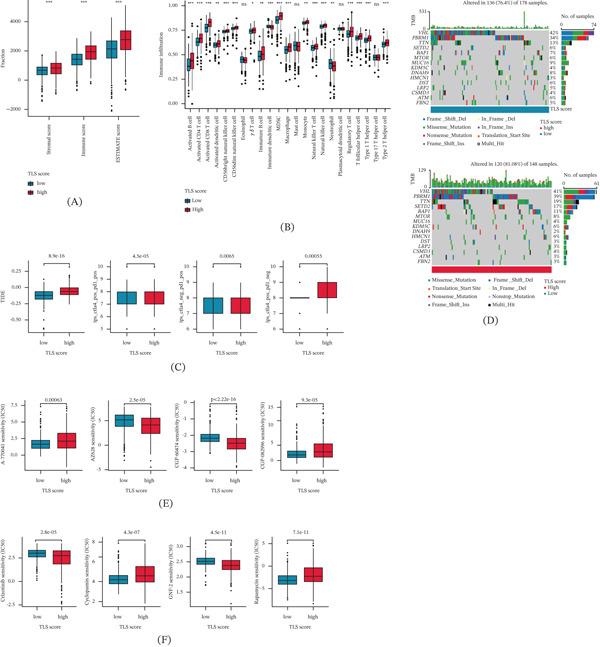
Therapeutic implications: Immunotherapy response and targeted agent prediction. (A) Immune status estimation. (B) Characterization of immune microenvironment of TLS score subtypes. (C) TIDE evaluation and IPS score of TLS score subtypes. Significance level: ∗*p* < 0.05, ∗∗*p* < 0.01, ∗∗∗*p* < 0.001. ns: no significance. (D) Genetic mutation frequency of TLS score subtypes. (E, F) Drug sensitivity prediction of TLS score subtypes.

The association of TLS score and immune microenvironment in ccRCC was further clarified based on ESTIMATE and ssGSEA algorithms. Immune status result indicated that the ccRCC samples with high TLS score were associated with higher estimate, immune, and stromal scores (Figure 4A). ssGSEA result illustrated that the high TLS score subtype was correlated with higher immune infiltration in most immune cells, such as activated B cell, CD4+ T cell, and CD8+ T cell (Figure 4B). The TIDE evaluation result illustrated that the ccRCC samples with low TLS score may be associated with better response to immunotherapy, and the IPS results revealed that the high TLS score subtype was more sensitive to the treatment of CTLA4 and PD1 (Figure 4C). These results demonstrate the potential association of TLS score and immune microenvironment characterization and could indicate the immunotherapy response of ccRCC samples in TLS score subtypes. Because the TLS score is constructed from immune‐related genes, associations between the score and immune infiltration metrics should be interpreted cautiously, as partial analytical dependence between variables may exist.

### 3.8. Genetic Mutation Landscape and Drug Sensitivity Analysis of TLS Score Subtype

We further explored the genetic mutation landscape of ccRCC in TLS score subtypes. Mutation characterization waterfalls results illustrated that the genetic mutation landscape was altered in 136 (76.4%) of 178 samples in low TLS score subtype and in 120 (81.08%) of 148 samples in high TLS score subtype. Compared to low TLS score subtype, the mutation frequencies of PBRM1 (39%), TTN (19%), SETD2 (17%), and BAP1 (11%) were higher in the high TLS score subtype (Figure 4D). By predicting of GDSC database, we obtained a series of antineoplastic drugs which may benefit the individualized treatment of ccRCC samples in TLS score subtypes. The IC50 of A‐770041, CGP‐082996, cyclopamine, and rapamycin was higher in the high TLS score subtype, whereas the low TLS score subtype was associated with higher IC50 of AZ628, CGP‐60474, crizotinib, and GNF‐2 (Figure 4E,F).

### 3.9. Histopathological Validation of TLS‐Derived Prognostic Biomarkers

**FIGURE 5:**
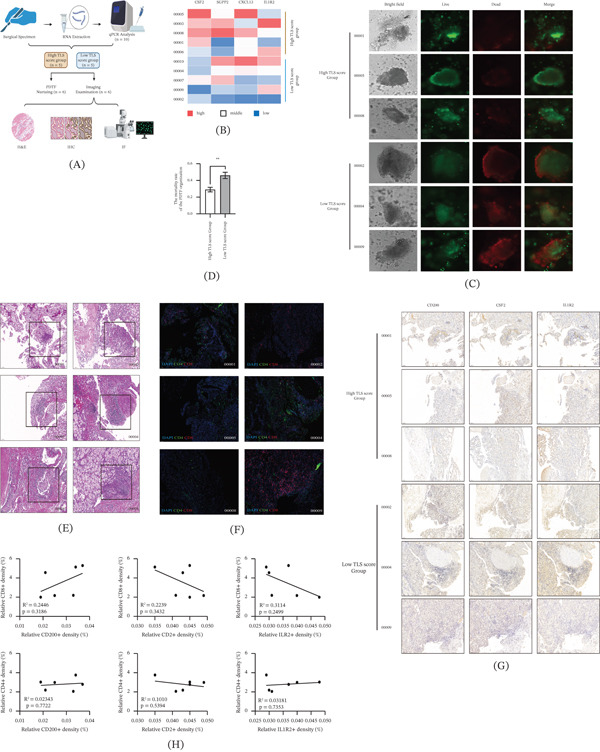
Experimental validation of TLS‐derived prognostic biomarkers in ccRCC. (A) Schematic workflow of experiments on 10 fresh ccRCC tissues: RNA extraction/qRT‐PCR for TLS score calculation, PDTF culture (*n* = 9, six qualified) with anti‐PD‐1 + anti‐CTLA‐4 treatment, and H&E/IF/IHC staining of six FFPE samples (4‐*μ*m sections). One sample was used only for RNA extraction due to insufficient volume. (B) Heatmap of relative mRNA expression of TLS signature genes (CSF2, SGPP2, CXCL13, and IL1R2) in 10 ccRCC samples (qRT‐PCR, GAPDH as reference). Samples ranked by TLS score (established formula); color intensity reflects expression level (red = high, blue = low). (C) Live/dead fluorescence images of PDTFs (six ccRCC samples) after 72‐h anti‐PD‐1 + anti‐CTLA‐4 treatment (Calcein‐AM: live cells, green; PI: dead cells, red). Bright field and merged images shown. Scale bars = 100 *μ*m. (D) Comparison of PDTF mortality (dead/total fluorescence intensity) between high and low TLS score groups. Data represent mean ± SD. ∗∗ indicates *p* < 0.01 as assessed by the Mann–Whitney *U* test. (E) H&E staining of 4‐*μ*m FFPE sections (six ccRCC samples); TLS regions (black arrows) identified by a pathologist. Scale bars = 200 *μ*m. (F) IF staining of TLS‐adjacent sections (six ccRCC samples): CD4+ (Alexa Fluor 488, green), CD8+ (Alexa Fluor 594, red) T cells, nuclei (DAPI, blue). Confocal images show T cell infiltration in TLS regions. Scale bars = 50 *μ*m. (G) IHC staining of TLS‐adjacent sections (six ccRCC samples) for CSF2/CD200/IL1R2 (1:1000 primary antibody dilution). DAB (brown) = positive expression; hematoxylin counterstain. Representative images for high/low TLS score groups. Scale bars = 100 *μ*m. (H) Correlation analysis of CSF2/CD200/IL1R2 protein density (IHC) versus CD8+/CD4+ T cell infiltration (IF) in six ccRCC samples (Pearson/Spearman).

To experimentally validate the clinical relevance of the TLS‐derived prognostic signature in ccRCC, we collected 10 fresh tumor tissue specimens from patients who underwent radical nephrectomy (Figure 5A). Total RNA was isolated from frozen tissues, and qRT‐PCR was performed to quantify the mRNA expression levels of four TLS signature genes (CSF2, CXCL13, IL1R2, and SGPP2), with glyceraldehyde‐3‐phosphate dehydrogenase (GAPDH) serving as the internal reference gene. The TLS score for each sample was calculated using the following formula: TLS Score = (CSF2 × 0.592) + (CXCL13 × 0.104) + (IL1R2 × 0.104) + (SGPP2 × (−0.325)), and samples were ranked according to their TLS scores (Figure 5B).

Primary PDTFs were cultured from nine of these samples (three were excluded due to poor growth), and six viable PDTF lines were treated with a combination of anti‐PD‐1 (nivolumab, 10 *μ*g/mL) and anti‐CTLA‐4 (ipilimumab, 10 *μ*g/mL) antibodies for 72 h to mimic first‐line clinical combination immunotherapy. Cell mortality was assessed via live/dead cell staining (Calcein‐AM for live cells, PI for dead cells), and fluorescence images were captured to visualize the distribution of live and dead cells (Figure 5C). Quantitative analysis demonstrated that PDTF mortality in the high TLS score group was higher than that in the low TLS score group, though the difference did not reach statistical significance (unpaired two‐tailed Student′s *t*‐test, *p* ≤ 0.05; Figure 5D).

For histopathological characterization, 4‐*μ*m‐thick formalin‐fixed paraffin‐embedded (FFPE) sections from the six qualified samples were subjected to H&E staining, and TLS regions were identified by an experienced pathologist under light microscopy (Figure 5E). Consecutive sections adjacent to the TLS regions were processed for IF staining to detect CD4+ (Alexa Fluor 488, green) and CD8+ (Alexa Fluor 594, red) T cell infiltration (Figure 5F) and IHC staining to evaluate the protein expression of CSF2, CD200, and IL1R2 in peri‐TLS areas (primary antibodies were diluted at 1:1000; Figure 5G).

Pearson/Spearman correlation analysis was performed to investigate the association between the relative protein density of CSF2/CD200/IL1R2 and the infiltration levels of CD8+ and CD4+ T cells. Notably, CD200 protein density was positively correlated with CD8+ T cell infiltration, whereas CSF2 and IL1R2 protein densities were negatively correlated with CD8+ T cell infiltration; no obvious directional associations were observed between the protein densities of CD200, CSF2, IL1R2, and CD4+ T cell infiltration (Figure 5H). Collectively, these histopathological and functional assays suggest that the TLS‐derived signature may correlate with immunotherapy sensitivity in ccRCC, and TLS‐associated molecules (CSF2, CD200, and IL1R2) may participate in regulating antitumor immunity by modulating CD8+ T cell infiltration within TLS regions, although these associations did not achieve statistical significance.

These findings provide robust histological confirmation that TLS‐derived biomarkers reflect distinct immune microenvironmental landscapes in ccRCC. Specifically, the negative correlations observed between CSF2/IL1R2 and CD8+ cytotoxic T cell density within TLS regions suggest that these molecules may contribute to local immunosuppressive mechanisms, potentially by impeding the recruitment, activation, or retention of effector T cells critical for antitumor immunity. In contrast, CD200′s positive association with CD8+ T cell infiltration points to its putative role in preserving or enhancing antitumor immune responses within TLS. Notably, no directional associations were detected between these TLS‐associated molecules and CD4+ T cell infiltration, implying that their immunomodulatory effects are likely specific to cytotoxic T cell subsets rather than pan‐T cell populations.

This multidisciplinary validation integrating qRT‐PCR–based TLS scoring, functional PDTF drug sensitivity assays, and spatial histopathological characterization reinforces the clinical relevance of our TLS‐based prognostic model for ccRCC. Beyond prognostic stratification, these results offer mechanistic insights into how TLS‐related genes shape patient outcomes: by fine‐tuning the composition and functional state of the immune microenvironment within and adjacent to TLS, these molecules may dictate the efficacy of immune checkpoint inhibition (e.g., anti‐PD‐1/CTLA‐4 combination therapy). While the observed correlations did not reach statistical significance likely due to the modest sample size (*n* = 6) used for histopathological analysis, they lay a critical foundation for larger, prospective studies to validate the functional role of CSF2, IL1R2, and CD200 in ccRCC immune modulation. Collectively, our data position TLS‐derived biomarkers as not only prognostic indicators but also potential therapeutic targets to rewire the tumor immune microenvironment and improve immunotherapy responses in ccRCC patients.

## 4. Discussion

In this study, we developed a transcriptional signature derived from TLS‐associated genes and explored its potential prognostic and immunological relevance in ccRCC. By integrating transcriptomic analysis of TCGA data with limited experimental observations from clinical specimens, our results suggest that TLS‐associated molecular patterns may capture aspects of immune heterogeneity in ccRCC.

The four‐gene TLS score constructed in this study (CSF2, CXCL13, IL1R2, and SGPP2) consistently stratified patients into groups with significantly different overall survival across the entire cohort as well as internal training and validation subsets. Multivariate analyses indicated that the TLS score remained independently associated with survival after adjustment for established clinical variables such as stage and grade. These results suggest that transcriptional patterns linked to TLS‐related immune processes may provide complementary prognostic information beyond conventional clinicopathological parameters.

An interesting observation of this study is that a higher TLS score was associated with poorer survival despite being accompanied by increased immune infiltration. At first glance, this finding appears inconsistent with the commonly reported association between mature TLS and favorable prognosis in several cancers. However, accumulating evidence indicates that immune infiltration alone does not necessarily translate into effective antitumor immunity. Instead, immune‐rich tumors may represent heterogeneous biological states, including productive immune responses, chronic inflammation, or immune exhaustion. Therefore, the TLS score identified in this study may reflect an immune‐active but functionally dysregulated or ineffective immune microenvironment rather than the presence of fully mature and protective TLS structures.

Importantly, the TLS score derived from bulk transcriptomic data should not be interpreted as a direct measurement of TLS presence or maturation. Bulk RNA sequencing captures averaged transcriptional signals from multiple cell populations within the TME. Consequently, the score likely reflects broader immune‐related transcriptional programs associated with TLS‐related genes rather than structural TLS formation itself. The TLS‐based molecular subtypes further supported the presence of immune heterogeneity. TLS cluster B exhibited relatively high immune infiltration yet poorer survival compared with cluster A. Such observations are consistent with the possibility that immune activation in ccRCC may coexist with immune suppression, exhaustion, or dysfunctional inflammatory signaling. Future studies incorporating spatial transcriptomics or single‐cell profiling may help clarify how TLS composition and maturation contribute to these distinct immune states.

Our analyses also explored the potential therapeutic implications of TLS‐associated transcriptional patterns. Computational prediction using TIDE and IPS algorithms suggested that TLS score subgroups may differ in their predicted responsiveness to immune checkpoint blockade. However, the interpretation of these results requires caution. Notably, TIDE and IPS analyses produced partially inconsistent predictions regarding immunotherapy responsiveness. This discrepancy likely reflects differences in the underlying modeling assumptions and datasets used by the two algorithms. Therefore, these results should be considered exploratory and hypothesis‐generating rather than direct evidence of treatment benefit. Similarly, predicted differences in drug sensitivity derived from the GDSC database represent in silico estimations based on gene expression profiles. These findings should not be interpreted as direct clinical recommendations but may instead provide hypotheses for future experimental investigation.

To provide preliminary biological context for the computational findings, we performed limited experimental validation using fresh ccRCC specimens. TLS score assessment by quantitative RT‐PCR was feasible in clinical samples, and spatial histological analyses suggested potential associations between TLS‐associated molecules and local immune cell infiltration. In particular, correlations between selected TLS‐associated proteins and CD8^+^ T cell density were observed in peri‐TLS regions. However, due to the modest sample size and the correlative nature of these analyses, no mechanistic conclusions can be drawn from these observations. The in vitro experiments using PDTFs treated with immune checkpoint antibodies were intended to provide exploratory context rather than to replicate the full complexity of clinical immunotherapy responses.

Several limitations of this study should be acknowledged. First, most analyses were based on retrospective TCGA datasets, which may introduce cohort‐specific biases. Second, the prognostic model was validated only through internal data splitting rather than an independent external cohort, which may increase the risk of overfitting. Third, the experimental validation cohort was relatively small and therefore had limited statistical power. Fourth, because the TLS score is constructed from immune‐related genes, associations between the score and immune infiltration measurements may partly reflect analytical dependence between variables. Finally, bulk transcriptomic data cannot directly resolve TLS structure or maturation state. Future studies incorporating spatial transcriptomics, single‐cell analysis, and larger prospective cohorts will be necessary to clarify the biological and clinical significance of TLS‐associated transcriptional programs in ccRCC.

In summary, our study identifies a TLS‐derived transcriptional signature that is associated with prognosis and immune microenvironment features in ccRCC. Rather than serving as a direct surrogate for TLS presence, the TLS score may capture broader immune context states within the TME. These findings highlight the potential value of TLS‐associated molecular patterns for understanding immune heterogeneity in ccRCC. Further validation in independent cohorts and mechanistic investigations will be necessary to determine whether TLS‐associated signatures can ultimately contribute to patient stratification or therapeutic decision‐making.

## 5. Conclusion

Our study demonstrates that a TLS‐derived gene signature is associated with prognosis and immune‐related features in ccRCC. By moving beyond a binary assessment of TLS presence, this approach highlights the relevance of TLS‐associated molecular heterogeneity in shaping clinical outcomes. While further validation is required, TLS‐based molecular profiling may represent a useful adjunct for prognostic assessment and exploratory immune stratification in ccRCC.

## Author Contributions

Xuanyu Zhou and Zhongwei Zhao contributed equally to this manuscript.

## Funding

This study was supported by the Natural Science Foundation of Shandong Province, 10.13039/501100007129, ZR2024QH044 and ZR2024QH065, and the China Postdoctoral Science Foundation, 10.13039/501100002858, 2024M761859.

## Conflicts of Interest

The authors declare no conflicts of interest.

## Data Availability

The data that support the findings of this study are available from the corresponding author upon reasonable request.
